# Altered Vagal Signaling and Its Pathophysiological Roles in Functional Dyspepsia

**DOI:** 10.3389/fnins.2022.858612

**Published:** 2022-04-22

**Authors:** Hui Li, Amanda J. Page

**Affiliations:** ^1^Vagal Afferent Research Group, Adelaide Medical School, The University of Adelaide, Adelaide, SA, Australia; ^2^Nutrition, Diabetes and Gut Health, Lifelong Health Theme, South Australian Health and Medical Research Institute, Adelaide, SA, Australia

**Keywords:** functional dyspepsia, vagus nerve, gut–brain axis, vagal afferent, vagal efferent

## Abstract

The vagus nerve is crucial in the bidirectional communication between the gut and the brain. It is involved in the modulation of a variety of gut and brain functions. Human studies indicate that the descending vagal signaling from the brain is impaired in functional dyspepsia. Growing evidence indicate that the vagal signaling from gut to brain may also be altered, due to the alteration of a variety of gut signals identified in this disorder. The pathophysiological roles of vagal signaling in functional dyspepsia is still largely unknown, although some studies suggested it may contribute to reduced food intake and gastric motility, increased psychological disorders and pain sensation, nausea and vomiting. Understanding the alteration in vagal signaling and its pathophysiological roles in functional dyspepsia may provide information for new potential therapeutic treatments of this disorder. In this review, we summarize and speculate possible alterations in vagal gut-to-brain and brain-to-gut signaling and the potential pathophysiological roles in functional dyspepsia.

## Introduction

Functional dyspepsia is a common upper gastrointestinal disorder affecting approximately 20% of the population. It is classified by Rome IV into three subtypes, PDS (accounting to 38% of functional dyspepsia patients), characterize by bothersome postprandial fullness and early satiation; EPS (27% of patients), characterized by gastric pain or burning; and a mixture of both (35% of patients) (Talley and Ford, 2015). Functional dyspepsia is a disorder of the gut–brain axis, with heterogeneous pathophysiological changes observed in both the upper gastrointestinal tract and the brain. The changes include gastroduodenal hypersensitivity (observed in 34–66% of patients), delayed gastric emptying (20–50% of patients), abnormal gastric accommodation (∼40% of patients), increased gastroduodenal inflammation and permeability (up to 40% of patients), and abnormal brain functions including abnormal psychological processes (Vanheel and Farre, 2013; Ford et al., 2020; Wauters et al., 2020b). The etiology of functional dyspepsia may arise either from brain to gut pathways, with psychological disorders occurring before gastric disorders, or from gut to brain pathways, with gastric disorders preceding the onset of psychological disorders (Talley, 2020). This bidirectional etiology suggests the gut–brain communication plays a critical role in the development of functional dyspepsia.

The vagus nerve is an important signaling pathway enabling communication between the gut and brain. The ascending vagal nerves (i.e., vagal afferents) sense local mechanical and chemical signals within the gut and send these signals to the brain. This is crucial for several principal brain functions, including the regulation of food intake and mood. The descending vagal nerves (i.e., vagal efferents) transfer brain signals to the gut, including brain signals triggered by stress or ascending gut signals (e.g., vagovagal reflex). These descending vagal efferent signals modulate gut functions including gastrointestinal sensation, gut motility, hormone secretion, acid secretion, and inflammation. Therefore, any alterations in vagal ascending signaling can have an impact on brain function and, further, on gut function, and any alterations in vagal descending signaling can have an impact on gut function and subsequently on brain function.

It is likely the vagus nerve is involved in the development of functional dyspepsia, given that the physiological functions of the vagus nerve largely overlap with the pathophysiological changes observed in functional dyspepsia. In addition, the bidirectional vagal signaling pathway is in accordance with the bidirectional etiological features of functional dyspepsia. Abundant evidence indicates a subset (∼30%) of functional dyspepsia patients have impaired vagal efferent activity (Hausken et al., 1993; Hveem et al., 1998; Lorena et al., 2002; Dal et al., 2014; Guo et al., 2018). In addition, recent findings, using animal models of functional dyspepsia, suggest vagal afferent signaling in response to gastric distension may be increased in functional dyspepsia (Li et al., 2019; Cordner et al., 2021). Furthermore, vagal nerve stimulation has been shown to reverse some of the pathophysiological changes (Hou et al., 2020; Zhu et al., 2021). This evidence suggests the vagus nerve could be a pathological component and a therapeutic target for functional dyspepsia, however, the role of vagal nerves are still largely unknown.

There is increasing knowledge of altered gut and brain signals in functional dyspepsia, providing potential implications for altered vagal signaling in the pathophysiology of this disorder. In this review, we summarize and speculate possible alterations in vagal gut-to-brain and brain-to-gut signaling and the potential pathophysiological implications in functional dyspepsia. We will particularly focus on the vagal innervation of the stomach and duodenum, the major gut regions associated with functional dyspepsia. It should be noted that there are parallel pathways in the communication between the gut and brain, e.g., *via* the blood circulation or sympathetic pathways or indirectly *via* vagal innervation of other organs, which should be recognized although beyond the scope of this review.

## Neuroanatomy of Vagal Neurocircuitry

The vagal neurocircuitry provides the structural foundations for the vagal mediated gut–brain communication. It is composed of vagal afferents, vagal efferents and the dorsal vagal complex in the brainstem, where vagal associated signals are integrated and coordinated centrally (Berthoud and Neuhuber, 2000).

### Vagal Afferent Nerves

The vagal afferent nerves constitute approximately 80-90% of the fibers in the vagus (Foley and DuBois, 1937; Asala and Bower, 1986). They have cell bodies located in the nodose ganglia and jugular ganglia, with nerve endings projecting both centrally and peripherally. In terms of vagal innervation of the gastrointestinal tract, there is a high density of peripheral endings in the gastroduodenal region, with a gradual reduction in density further down the gastrointestinal tract (Wang and Powley, 2000). Vagal afferent gastroduodenal terminals can be functionally classified into mechanosensitive and chemosensitive afferents. Mechanosensitive vagal afferents sense mechanical stimuli including food related mucosal stroking, distension and contraction. It includes mechanosensitive mucosal endings, IGLEs located in the myenteric plexus between the muscular layers, and IMAs, distributed within the muscular layers (Clarke and Davison, 1978; Zagorodnyuk et al., 2001). Chemosensitive vagal afferents have peripheral endings which sense chemical stimuli including nutrients, hormones, pH, and immune stimuli (pathogen, food allergies, and microbiota). The central endings of gastroduodenal vagal afferents terminate in the dorsal vagal complex in the brainstem, mostly in the NTS, but also in the area postrema, the DMV and the trigeminal island (Kalia and Sullivan, 1982).

### Dorsal Vagal Complex

The dorsal vagal complex consists of the NTS, DMV, area postrema, and the trigeminal island. It receives ascending gastroduodenal vagal afferent input. The neurons in the NTS have ascending axons projecting to a large number of brain areas, including brain areas involved in food intake regulation and mood, such as the hypothalamus and amygdala (Ricardo and Koh, 1978; Rinaman, 2007, 2010). The NTS neurons also have nerve fibers projecting directly or indirectly to vagal efferent neurons in the DMV, constituting a pathway for reflex feedback to the gastrointestinal tract, *via* the vagal efferent pathway, to modulate gut functions. In addition, the dorsal vagal complex receives descending projections from higher brain regions, including the cortex, hypothalamus and amygdala (van der Kooy et al., 1984). The dorsal vagal complex can also be readily modulated by circulating factors, including hormones and inflammatory factors (Blevins et al., 2000), as it lies outside the blood–brain barrier.

### Vagal Efferent Nerves

The vagal efferent nerves account for approximately 10–20% of vagal fibers (Foley and DuBois, 1937; Asala and Bower, 1986). The cell bodies of vagal efferent nerves innervating the stomach and duodenum are located within the DMV, descending mainly *via* the gastric and hepatic branches of the vagus nerve (Berthoud et al., 1991). Their axons project to gastroduodenal myenteric neurons in the myenteric plexus of the enteric nerve system (Berthoud et al., 1991). Vagal efferent mediated activation of myenteric neurons leads to the regulation of gastrointestinal functions, such as motility, secretion, and immune responses (Chang et al., 2003).

## Vagal Afferent Sensation of Gastroduodenal Mechanical Stimuli in Functional Dyspepsia

There is a recent review on the neural signaling of gut mechanosensation in ingestive and digestive process which provides a comprehensive review of gut mechanosensation (Kim et al., 2022). In the gastroduodenal region, mechanosensitive vagal afferents sense gastroduodenal distension (Zagorodnyuk and Brookes, 2000) and generate signals in linear relation to the levels of distension, i.e., the degree of filling of the gastroduodenal lumen (McCann and Rogers, 1992). Besides distension, muscle contraction can also activate tension sensitive vagal afferents (Iggo, 1955, 1957), and contraction can further enhance the response of vagal afferents to distension (Iggo, 1955). Evidence indicates the distension and contraction signals may be sensed by IGLE terminals in the gastroduodenal region. More specifically, studies indicate distinct populations of glucagon-like peptide 1 receptor (GLP-1R) expressing IGLEs in the stomach and oxytocin receptor (Oxtr) expressing IGLEs in the duodenum are the major tension sensitive vagal afferents (Williams et al., 2016; Bai et al., 2019). These signals are conveyed to the brain to regulate food intake, mood and indirectly pain sensation, or transferred *via* the vagal reflex pathway to modulate gastric motility and secretion. In functional dyspepsia, gastroduodenal mechanical stimuli are altered (Troncon et al., 1994) and the sensitivity of vagal afferents to mechanical stimuli is increased (Li et al., 2019; Cordner et al., 2021), which likely leads to altered vagal afferent signaling and corresponding gut and brain malfunctions.

### Altered Gastroduodenal Mechanical Stimuli in Functional Dyspepsia

Evidence indicates food induced gastroduodenal distension may be altered in functional dyspepsia. A subset of functional dyspepsia patients have abnormal intragastric distribution of food, with reduced food accommodation in the proximal stomach, increased antral area (Hausken and Berstad, 1992; Pilichiewicz et al., 2008) and food retention in the antrum (Troncon et al., 1994; Urbain et al., 1995). Therefore, in these patients, the level of food induced distension may be reduced in the proximal stomach and enhanced in the gastric antrum, hence reducing or increasing the corresponding ascending distension sensitive vagal afferent signals. Furthermore, functional dyspepsia is associated with an increase in gastric antral contractions (antral motor activity indices) after food intake (Urbain et al., 1995), ultimately leading to increased activation of mechanosensitive vagal afferents.

### Increased Vagal Afferent Sensitivity to Gastric Distension in Functional Dyspepsia

Recent animal studies indicate the sensitivity of gastric vagal afferents to gastric distension is increased in functional dyspepsia (Li et al., 2019; Cordner et al., 2021), consistent with the increased sensitivity to gastric distension in functional dyspepsia patients (Coffin et al., 1994; Barbera et al., 1995a; Caldarella et al., 2003). It should be noted that this vagal afferent hypersensitivity has been consistently observed in two different animal models of functional dyspepsia, namely an unpredictable chronic mild stress mouse model (Li et al., 2019), which has a central origin, and a gastric irritation (0.1% iodoacetamide) induced rat model, which has a peripheral origin within the gut (Cordner et al., 2021). This suggests vagal afferent hypersensitivity to gastric distension may be a crucial pathological component of functional dyspepsia with both etiologic origins. Human studies have also shown that functional dyspepsia patients have increased perception to balloon-induced duodenal distension (Holtmann et al., 1996). Whether this involves hypersensitivity of duodenal vagal afferents to duodenal distension requires further investigation.

The sensitivity of mechanosensitive vagal afferents can be modulated by a variety of circulating or locally released mediators, including hormones, neurotransmitters, inflammatory factors, endotoxins and pH. Therefore, altered levels of these mediators or altered modulation by these mediators may lead to vagal afferent hypersensitivity. For example, the gut hormone CCK, released from the duodenum, has been shown to increase the sensitivity of gastric and duodenal vagal afferents to distension (Davison and Clarke, 1988; Blackshaw and Grundy, 1990; Schwartz et al., 1995). In functional dyspepsia, diet induced CCK release is increased (Pilichiewicz et al., 2008), and the CCK-A receptor antagonist dexloxiglumide reduced gastric distension-induced symptoms in functional dyspepsia patients (Feinle et al., 2001). Therefore, it is possible that vagal afferent distension signals are enhanced due to the increase in CCK levels, leading to functional dyspepsia symptoms. Further, in functional dyspepsia, specifically patients with PDS, circulating acylated ghrelin levels are reduced (Shindo et al., 2009; Choi et al., 2016). Ghrelin is known to inhibit vagal afferent responses to gastric distension (Page et al., 2007) and, therefore, a reduction in ghrelin levels is likely to lead to an increase in sensitivity of gastric vagal afferents. As these afferents play an important role in satiety signaling (Date et al., 2002), it is conceivable that this pathway could contribute to the symptoms of early satiety and/or bloating observed in PDS, however, there is a lack of direct evidence. Furthermore, chemical stimuli such as hydrochloric acid also increase the response of gastric vagal afferents to distension (Kang et al., 2004). In functional dyspepsia although the rate of gastric acid secretion was within the normal range (Collen and Loebenberg, 1989), duodenal acid exposure was increased in a subgroup (64%) of patients (Lee et al., 2004), likely as a consequence of the reduced duodenal acid clearance rate (Samsom et al., 1999). This increase in luminal acid exposure may contribute to increased vagal afferent mechanosensitivity in a subgroup of functional dyspepsia patients.

Evidence indicates inflammation, specifically mast cell infiltration, may play a role in vagal afferent hypersensitivity. Mast cell infiltration has been observed in the stomach and duodenum of functional dyspepsia patients (Du et al., 2018) and can be triggered by both stress and gastric irritation in animal models (Cordner et al., 2021). In a gastric irritation-induced rat model of functional dyspepsia, the mast cell stabilizer ketotifen reduced vagal afferent hypersensitivity as well as the associated pain response and psychological disorders (Cordner et al., 2021). A variety of molecular mediators are released by mast cells in response to environmental changes, including reactive oxygen species, histamine, serotonin, protease, and cytokines (Traina, 2019), however, their effects on mechanosensitive vagal afferent are unknown and require investigation. In addition, whether other immune cells, such as eosinophils, are also involved in this vagal afferent hypersensitivity is not known. Identification of the molecular factors contributing to vagal afferent hypersensitivity to food related stimuli (e.g., stretch) may provide novel new therapeutic targets to improve the symptoms associated with functional dyspepsia.

### Pathophysiological Roles of Enhanced Vagal Signaling in Response to Distension in Functional Dyspepsia

**FIGURE 1:**
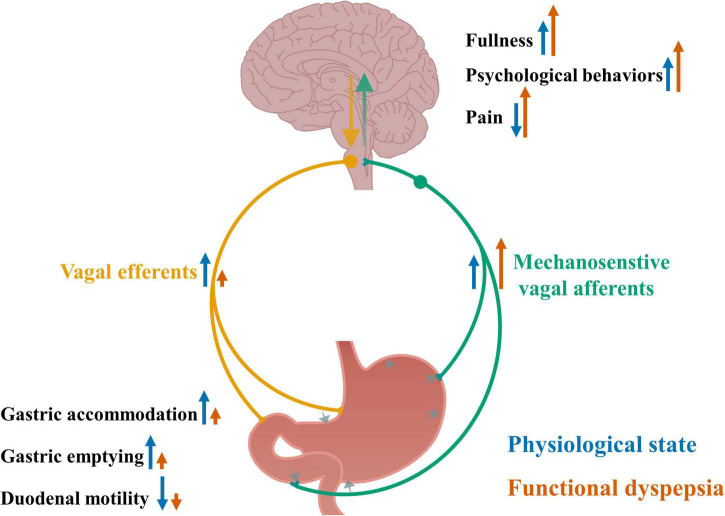
Altered vagal signaling in response to gastric distension and its associated pathophysiological changes in functional dyspepsia. Gastric distension and contraction activate mechanosensitive vagal afferents and send signals to the brain, leading to increased feeling of fullness, increased psychological behaviors and reduced pain sensation, and further *via* vagal efferents send signals to the gut, leading to increased gastric accommodation and emptying, and reduced duodenal motility. In functional dyspepsia, the sensitivity of vagal afferents to gastric distension is increased, accompanying increased feeling of fullness, psychological behaviors and pain, and impaired gastric accommodation and emptying, and duodenal motility. Blue arrows indicate an effect to increase or decrease nerve activity or physiological outcomes under normal physiological conditions. Orange arrows indicate an effect to increase or decrease nerve activity or physiological outcomes in functional dyspepsia and the size of the arrows indicate the levels of the effects.

Increased vagal signaling in response to distension may be associated with multiple pathophysiological changes and symptoms in functional dyspepsia, including altered food intake, mood, pain perception, gastric accommodation and emptying and duodenal motility, which are summarized in Figure 1.

#### In the Modulation of Food Intake

Detection of gastric distension by gastric vagal afferents is a well-appreciated physiological mechanism for the initiation of postprandial satiation and termination of food intake. The level of antral distension is positively correlated with postprandial fullness in healthy subjects (Jones et al., 1997). A recent study found that duodenal vagal afferent responses to duodenal distension also contribute to the postprandial inhibition of food intake, providing a more sustained inhibition of food intake compared to gastric afferents (Bai et al., 2019). The inhibition of food intake in response to vagal afferent distension signals may involve multiple regions in the hypothalamus, the central center for appetite regulation. For example, activation of gastric and duodenal tension sensitive vagal afferents led to inhibition of AgRP neurons in the hypothalamus and reduced food intake in mice (Bai et al., 2019). Furthermore, vagal afferent responses to gastric distension activate oxytocin secreting neurons in the PVN to release oxytocin in rats (Ueta et al., 1991), which is known to inhibit food intake *via* multiple central pathways (Onaka and Takayanagi, 2019).

Functional dyspepsia, specifically PDS, is characterized by bothersome postprandial fullness and early satiety. In functional dyspepsia patients, delayed gastric emptying, resulting in increased gastric distension, was found to be associated with symptom severity of early satiety and postprandial fullness (Stanghellini et al., 1996; Cuomo et al., 2001). In addition, impaired gastric accommodation in functional dyspepsia, likely increasing the response of mechanosensitive vagal afferents to the same amount of food, was also associated with early satiety and weight loss in functional dyspepsia, with normalization of gastric accommodation improving meal-induced early satiety (Tack et al., 1998). Therefore, it is possible that mechanosensitive vagal afferents are involved in the impaired gastric emptying and impaired gastric accommodation induced postprandial fullness and early satiety in functional dyspepsia. Furthermore, there is also direct evidence to indicate vagal afferents are hypersensitive to gastric distension, in an unpredictable chronic mild stress mouse model of functional dyspepsia, which is accompanied by reduced food intake and body weight (Li et al., 2019). This further supports the possibility that vagal afferent hypersensitivity may lead to reduced food intake. Therefore, there are likely a number of additive factors that contribute to the hypersensitivity observed in functional dyspepsia, with any combination of these factors leading to similar symptoms, reinforcing the idea of personalized treatment. The factors contributing to hypersensitivity of the gut–brain axis requires further investigation before we can consider this type of targeted therapy. Despite the importance of distension sensitive duodenal vagal afferent in initiating satiation, the role of duodenal afferents in functional dyspepsia has not been investigated.

#### In the Modulation of Mood

Evidence indicates vagal sensation of gut signals is involved in the modulation of mood and emotion. In healthy rats, complete and selective subdiaphragmatic vagal deafferentation, which block vagal sensation of abdominal signals including gut signals, reduced innate anxiety-like behavior and learned fear (Klarer et al., 2014). This is associated with altered neurotransmitter levels, i.e., increased GABA and decreased noradrenaline in specific regions of the limbic area, the critical area regulating both innate anxiety and conditioned fear (Klarer et al., 2014). Furthermore, optical activation of the gut-innervating vagal sensory neurons in the right ganglion of mice stimulated reward behaviors and induced dopamine release from the substantia nigra (Han et al., 2018). This suggests vagal sensation of gut signals may play a role in the modulation of emotional states.

Functional dyspepsia is highly associated with psychological disorders, including anxiety and depression. In some patients, the psychological disorders occur after the gastric symptoms, suggesting altered gut signals may contribute to the development of these psychological disorders (Talley, 2020). In gastric irritation-induced functional dyspepsia rats, vagal afferent hypersensitivity to gastric distension accompanied increased anxiety- and depressive-like behaviors (Cordner et al., 2021). Furthermore, subdiaphragmatic vagotomy or reversing vagal afferent hypersensitivity, using the mast cell stabilizer ketotifen, normalized these psychological behaviors (Cordner et al., 2021). This suggests the vagal afferent hypersensitivity to gastric distension may lead to psychological disorders. Consistent with this, rat vagal afferent deafferentation reduced innate anxiety-like behavior and learned fear in physiological conditions (Klarer et al., 2014). Furthermore, in a rat model of functional dyspepsia vagal afferent hypersensitivity was accompanied by an increase and decrease in gene expression of CRF and BDNF respectively in the amygdala, with the altered expression partially normalized by subdiaphragmatic vagotomy (Cordner et al., 2021). Since increased CRF and decreased BDNF have been observed in increased anxiety- and depressive-like behavior (Liu et al., 2011; Sagarkar et al., 2017), the altered gene expression may contribute to the central mechanisms responsible for vagal afferent induced psychological disorders in functional dyspepsia.

#### In the Modulation of Pain Perception

There is growing evidence to indicate vagal afferent signaling may indirectly modulate pain sensation. For example, activation of gastric vagal afferents through non-painful gastric distension reduced somatic pain perception in healthy human (Sedan et al., 2005). In addition, vagal nerve stimulation has been shown to relieve pain in both human and animal studies, as summarized in a review paper by Chakravarthy et al. (2015), possibly *via* a mechanism involving activation of vagal afferents. The anti-nociceptive effects of vagal nerve stimulation may be *via* different pathways, including inhibition of nociceptive neurons in the spinal cord (Ammons et al., 1983; Chandler et al., 1991), activation of the vagal anti-inflammatory pathway which inhibits inflammation associated pain (Hosoi et al., 2000; Khasar et al., 2003), modulation of attention and mood pathways which interact with the pain sensation pathway(George et al., 2000), or modulating other brain areas that modulate pain (Randich and Aicher, 1988; Nishikawa et al., 1999).

Functional dyspepsia, specifically EPS is characterized by epigastric pain or burning. Hypersensitivity to gastroduodenal distension is well described to induce pain in functional dyspepsia (Holtmann et al., 1996; Tack et al., 2001) and evidence suggests this may involve a vagal afferent pathway. In a rat model of functional dyspepsia, gastric pain accompanied vagal afferent hypersensitivity to gastric distension (Cordner et al., 2021). This pain behavior was partially improved by vagotomy or when vagal afferent hypersensitivity was reversed (Cordner et al., 2021). This suggests a role of vagal distension signaling in the increased pain perception observed in functional dyspepsia, which is in contrast to the anti-nociceptive effects observed in physiological conditions. Indeed, the gastric distension-induced pain behavior in rats with functional dyspepsia was attenuated by blocking ventrolateral PAG activity, the central descending pathway through which vagal afferents can convey information to the spinal cord (Craig, 2003), suggesting vagal hypersensitivity may increase gastric pain through the activation of brain descending signals to the spinal cord (Cordner et al., 2021). In addition, in functional dyspepsia patients, gastric distension failed to activate the brain area pregenual anterior cingulate cortex (pACC) and brain signals known to inhibit anxiety (Van Oudenhove et al., 2010). Anxiety is known to exacerbate pain sensation, therefore, the impaired modulation of gastric distension on anxiety may also lead to increased pain sensation.

#### In the Modulation of Gastroduodenal Motility

The response of gastroduodenal vagal afferents to distension is crucial for the regulation of gastroduodenal motility, i.e., relaxation and contraction, *via* a vagovagal reflex. The vagal reflex pathway includes: (1) gastric distension (corpus and antrum)-induced relaxation of the proximal stomach (Abrahamsson, 1973; Abrahamsson and Jansson, 1973), which allows accommodation of ingested food without significantly increasing intragastric pressure; (2) antral distension-induced relaxation of the pylorus and inhibition of pyloric contractions, which enables the pylorus to receive food from the antrum and facilitate gastric emptying into the duodenum (Ishiguchi et al., 2001); and (3) duodenal distension-induced relaxation of the gastric corpus and antrum and contraction of the pylorus (De Ponti et al., 1987; Holzer and Raybould, 1992; Coffin et al., 1994; Shafik, 1998), which prolongs food digestion in the stomach and delays gastric emptying.

Population studies indicate gastric distension-induced gastric relaxation, duodenal distension-induced gastric relaxation (Coffin et al., 1994), and duodenal distension-induced inhibition of small intestinal motility are impaired in functional dyspepsia (Holtmann et al., 1996), suggesting an impaired vagovagal reflex likely contributes to the impaired gastric accommodation, delayed gastric emptying and reduced duodenal motility in functional dyspepsia (Ford et al., 2020). The direct role of vagal afferents in these pathophysiological changes has not been reported, however, the increased vagal afferent signals in response to gastric distension [i.e., vagal afferent hypersensitivity, observed in mouse models of functional dyspepsia (Li et al., 2019; Cordner et al., 2021)], should amplify the vagovagal reflex and, therefore, it is unlikely abnormal vagal afferent signaling contributes to the impaired vagovagal reflex in functional dyspepsia. Other mechanisms, such as altered central processing of vagal afferent signals, and/or descending vagal efferent signaling, may be involved, which requires further investigation.

## Vagal Afferent Sensation of Gastroduodenal Nutrients, Hormones and pH Levels in Functional Dyspepsia

Gastroduodenal chemosensitive vagal afferents can sense chemical stimuli including nutrients, gut hormones and pH levels. Chemosensitive vagal afferents, mainly in the duodenum, have been found to detect and respond to a wide range of nutrients, including amino acids (Jeanningros, 1982), short-chain fatty acids (Lal et al., 2001), and glucose (Mei, 1978). The nutrient-induced activation of vagal afferents is mainly secondary to nutrient-induced gut hormone release, such as CCK activation of duodenal vagal afferents (Feinle et al., 1996; Richards et al., 1996; Reidelberger et al., 2004), serotonin activation of duodenal vagal afferents (Zhu et al., 2001), GLP-1 activation of gastric vagal afferents (Bucinskaite et al., 2009) and PYY activation of gastric vagal afferents (Abbott et al., 2005; Koda et al., 2005), although direct nutrient-induced activation has also been observed (Grabauskas et al., 2010). Capsaicin, a chemical compound found in some foods, has been shown to activate gastroduodenal chemosensitive vagal afferents (Blackshaw et al., 2000). These vagal signals are conveyed to the brain, involved in the modulation of food intake, nociception, or gastric motility and secretion. Luminal acid has been found to activate gastric vagal afferents (Richards et al., 1996). The activation of vagal afferents, by noxious levels of acid, leads to activation of several brain areas, including amygdala, hypothalamic PVN, supraoptic nucleus, habenula, involved in the regulation of mood, nociception, emotion, and food intake (Michl et al., 2001). Further, there is evidence to indicate that vagal afferent responses to acid contribute to acid-induced pain sensation (Lamb et al., 2003).

### Increased Gastroduodenal Food Chemicals and Hormones, and Reduced pH Levels in Functional Dyspepsia

**FIGURE 2:**
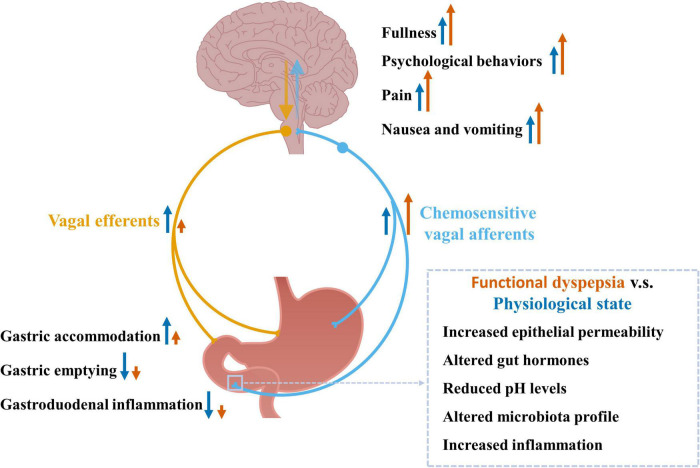
Chemosensitive vagal afferent signaling in response to altered chemical stimuli observed in functional dyspepsia and the associated pathophysiological changes. Chemosensitive vagal afferents can sense chemical stimuli including nutrients, gut hormones, pH levels, and inflammation-associated factors. These signals are sent to the brain *via* vagal afferents where the information is processed leading to increased fullness, psychological behaviors, pain sensation, nausea, and vomiting, as well as reflex signals back to the gut, *via* vagal efferents, leading to increased gastric accommodation and reduced gastric emptying. Functional dyspepsia is associated with increased gastroduodenal permeability, infiltration of eosinophil and mast cells, altered microbiota profile and increased chemical stimuli, which may enhance vagal afferent signals to the brain contributing to the increased feelings of fullness, psychological behaviors, pain sensation, nausea and vomiting observed in functional dyspepsia. The chemical stimuli induced vagovagal reflex is impaired in functional dyspepsia (Feinle et al., 2001; Schwartz et al., 2001), which may lead to impaired gastric accommodation, and impaired inhibition on gastric emptying and gastroduodenal inflammation.

Functional dyspepsia patients may have increased exposure of gastroduodenal chemical stimuli. Firstly, functional dyspepsia is associated with increased small intestinal mucosal permeability (Vanheel et al., 2014). This ‘leaky’ gut may increase the passive diffusion of luminal content into the mucosa, such as the food chemical capsaicin and luminal acid, therefore increasing their direct contact with chemosensitive vagal afferents in the mucosa. In addition, the production of gut hormones could be altered in functional dyspepsia, contributing to their altered exposure to vagal afferents. For example, lipid-induced release of CCK is upregulated in functional dyspepsia (Pilichiewicz et al., 2008). Furthermore, despite normal gastric fluid acid levels (Collen and Loebenberg, 1989), functional dyspepsia patients have increased exposure of acid in the duodenum (Lee et al., 2004), probably due to delayed clearance of acid from the duodenum (Samsom et al., 1999). This, in combination with a ‘leaky’ gut, may increase the exposure of acid to vagal afferent endings. The impact of altered chemical stimuli within the gut on chemosensitive vagal afferents in functional dyspepsia and the potential pathophysiological changes are illustrated in Figure 2.

### Hypersensitivity of Chemosensitive Vagal Afferents in Functional Dyspepsia

There is no direct evidence on whether the sensitivity of vagal afferents to chemical stimuli is altered in functional dyspepsia. However, there is abundant evidence that functional dyspepsia patients have increased symptoms in response to exposure of several chemical stimuli. In functional dyspepsia patients, intraduodenal lipid infusion induces symptoms, including bloating, fullness, discomfort, nausea, and vomiting (Barbera et al., 1995a,b). A subgroup of functional dyspepsia patients demonstrate hypersensitivity to capsaicin ingestion, with significantly higher gastrointestinal symptoms compared to healthy controls (Hammer et al., 2008; Fuhrer et al., 2011; Hammer and Fuhrer, 2017). In addition, CCK administration triggers symptoms, including pain, early satiety, abdominal bloating, nausea, and vomiting, in functional dyspepsia patients (Chua et al., 1994). Furthermore, functional dyspepsia patients have increased nausea in response to duodenal acid infusion compared with healthy subjects (Samsom et al., 1999; Lee et al., 2004). Overall, this evidence suggests that hypersensitivity to chemical stimuli exists in functional dyspepsia, which may involve the hypersensitivity of chemosensitive vagal afferents, however, this requires further investigation.

### Pathophysiological Roles of Altered Vagal Signaling in Response to Chemical Stimuli in Functional Dyspepsia

#### In the Modulation of Food Intake

Functional dyspepsia patients have heightened feelings of fullness in response to intraduodenal infusion of lipid compared to healthy controls (Barbera et al., 1995a,b). The lipid-induced early satiety could be due to an increase in lipid-induced CCK release (Feinle et al., 1996). CCK is known to inhibit food intake *via* the activation of chemosensitive vagal afferents and, in functional dyspepsia, high fat diet-induced CCK release is increased (Pilichiewicz et al., 2008). Further, CCK administration enhanced feelings of fullness and abdominal bloating (Chua et al., 1994) and infusion of CCK antagonists reduced lipid-induced feelings of fullness (Feinle et al., 2001). This suggests increased CCK signaling, *via* chemosensitive vagal afferents, may contribute to the early satiety observed in functional dyspepsia, although other pathways such as the modulatory effect of CCK on mechanosensitive vagal afferents could also be altered in functional dyspepsia. PYY is another anorexigenic hormone which can inhibit food intake *via* the activation of vagal afferents. Although postprandial PYY levels are reported to be reduced (Pilichiewicz et al., 2008) or unchanged (Witte et al., 2016) compared to healthy control, there is a correlation between postprandial PYY levels and feelings of fullness, suggesting a potential role of this hormone in early satiety in functional dyspepsia (Witte et al., 2016). In functional dyspepsia, postprandial GLP-1 levels are not changed and not associated with sensation of fullness (Witte et al., 2016). In addition, although serotonin inhibits food intake *via* vagal afferents, it is reported plasma serotonin levels are reduced (Cheung et al., 2013) or not changed (Faure et al., 2010) in functional dyspepsia, suggesting it may not contribute to the increased perception of fullness in functional dyspepsia, however, direct evidence is required.

#### In the Modulation of Pain Perception, Nausea, and Vomiting

Most functional dyspepsia patients have increased sensitivity to duodenal lipid infusion, which induces discomfort, pain, nausea, and vomit (Barbera et al., 1995a; Feinle-Bisset et al., 2003). These nutrient-induced symptoms are specific to fat, and not observed with glucose (Barbera et al., 1995b; Pilichiewicz et al., 2008). These lipid-induced symptoms may, at least in part, be mediated *via* lipid-induced CCK release. In functional dyspepsia patients, plasma CCK concentrations are positively correlated with the scores for nausea and pain (Pilichiewicz et al., 2008). Furthermore, in functional dyspepsia, intravenous administration of CCK increases abdominal pain, nausea, and vomiting (Chua et al., 1994). Further, the discomfort and nausea induced by lipid infusion are alleviated by treatment with the CCK-1 receptor antagonist dexloxiglumide (Feinle et al., 2001). Although serotonin induces nausea and vomiting *via* vagal afferents, in functional dyspepsia patients plasma serotonin levels are reduced (Cheung et al., 2013), suggesting it may not play an important role in nausea and vomiting in functional dyspepsia. In functional dyspepsia patients, postprandial GLP-1 levels are not changed, however, a positive correlation of GLP-1 levels with nausea was observed, suggesting a potential role of GLP-1 in nausea in functional dyspepsia (Witte et al., 2016). Evidence indicates duodenal acid plays a more significant role in inducing nausea in functional dyspepsia patients, compared to lipid and carbohydrate (Schwartz et al., 2001; Lee et al., 2004). In addition, intragastric acid administration also triggered the pain response, *via* vagal afferents, in rats (Lamb et al., 2003). In functional dyspepsia, duodenal acid exposure is increased (Samsom et al., 1999), which may trigger nausea and pain *via* a vagal afferent pathway.

#### In the Modulation of Gastroduodenal Motility

A subgroup of functional dyspepsia patients have reduced gastric compliance and/or delayed gastric emptying (Ford et al., 2020). Evidence indicates this may, at least in part, be due to impaired modulation of nutrients on gastric relaxation and motility. In functional dyspepsia patients, lipid-induced gastric compliance was lower compared to the healthy control group and, furthermore, the CCK-1 receptor antagonist dexloxiglumide blocked lipid-induced gastric compliance (Feinle et al., 2001). Therefore, it is possible that the lipid–CCK–vagal pathway may be altered in functional dyspepsia, contributing to impaired gastric compliance and delayed gastric emptying. In addition, functional dyspepsia is also associated with impaired duodenal motility (Sha et al., 2009). Evidence indicates this could be due to a reduction in acid-induced duodenal motility in functional dyspepsia patients (Schwartz et al., 2001). Whether this involves an impaired acid-vagal pathway requires investigation.

## Vagal Afferent Sensation of Gastroduodenal Immune Stimuli in Functional Dyspepsia

The lumen of the gastrointestinal tract can be exposed to external harmful stimuli, which is defensed by mucosal structural barriers, as well as a local immune system including immune cells. This local immune defense system is stimulated by luminal antigens, pathogens, microbiota, and lipid (known as postprandial low-grade inflammation). Vagal afferents are important immunosensory nerves in the gastrointestinal tract, which can be directly activated by immune stimuli, such as LPS (Hosoi et al., 2005), or indirectly through immune stimuli associated release of: (1) microbiota products, such as short chain fatty acids; (2) hormones [e.g., serotonin released in response to LPS or short chain fatty acids (Dalile et al., 2019) and CCK released in response to long chain fatty acids (Lal et al., 2001), or IL-1β (Kurosawa et al., 1997)]; or (3) inflammatory mediators, such as cytokines released from immune cells (Steinberg et al., 2016; Zanos et al., 2018).

Immune cells can be activated by antigens and release a variety of mediators. For example, mast cells have been shown to release histamine, serotonin, tryptase, and cytokines (TNF, IL-4, basic fibroblast growth factor, stem cell factor) (Moon et al., 2014), and eosinophils release IL-12, IL-4, transforming growth factor-β, chemokines, and growth factors (Travers and Rothenberg, 2015) in response to immune stimuli. Vagal afferent endings are in close contact with mast cells in the small intestinal mucosa (Williams et al., 1997) and receptors for some of these mediators, such as 5-HT3, TNF and IL-1β, have been shown to be expressed in vagal afferent neurons (Steinberg et al., 2016). Further, these mediators have been shown to activate vagal afferents (Hillsley and Grundy, 1998; Steinberg et al., 2016; Zanos et al., 2018). These vagal mediated immune signals contribute to multiple functions, such as peripheral IL-1 and LPS-induced depression (Bluthe et al., 1994, 1996; Luheshi et al., 2000), IL-1β and LPS-induced inhibition of food-motivated behavior (Bret-Dibat et al., 1995), endotoxin, IL-1 and TNF-induced hyperalgesia (Watkins et al., 1994, 1995), and bacterial endotoxin, TNF and IL-1β-induced activation of the HPA axis (Gaykema et al., 1995; Fleshner et al., 1998).

### Enhanced Gastroduodenal Immune Signals in Functional Dyspepsia

A subset of functional dyspepsia patients is associated with increased gastroduodenal permeability, which can increase the passive diffusion of luminal immune stimuli into the gut (Vanheel et al., 2014). Several studies have demonstrated that the gastroduodenal microbiota composition and amount are different between functional dyspepsia patients and healthy subjects, and the microbiota dysbiosis may be associated with the severity of functional dyspepsia symptoms, which have been well summarized in recent reviews (Tziatzios et al., 2020; Wauters et al., 2020a; Gurusamy et al., 2021). In addition, it is reported that ∼17% of functional dyspepsia patients have symptoms initiated after acute gastroenteritis (Tack et al., 2002). Several pathogens, including *Salmonella* spp., *Escherichia coli O157*, *C. jejuni*, *Giardia lamblia*, and *Norovirus* are associated with the post-infectious functional dyspepsia symptoms (Futagami et al., 2015). A subgroup of functional dyspepsia patients have gastroduodenal low-grade inflammation, with increased mast cell and eosinophil infiltration and degranulation. In *H. pylori* negative functional dyspepsia patients, plasma cytokines IL-1β, IL-10, and TNF-α levels are increased compared to healthy subjects (Liebregts et al., 2011). Furthermore, duodenal transcript expression of IL-1β, but not TNF-α is increased in functional dyspepsia (Komori et al., 2019). Indeed, plasma cytokine IL-1β, IL-10, and TNF-α levels are correlated with symptom intensity of pain, cramps, nausea, and vomiting, and associated with delayed gastric emptying, in *H. pylori* negative functional dyspepsia patients (Liebregts et al., 2011). Therefore, immune signals sensed by vagal afferents may be amplified, which may be involved in the corresponding symptoms, as illustrated in Figure 2.

### Pathophysiological Roles of Altered Vagal Signaling in Response to Gastroduodenal Inflammation in Functional Dyspepsia

#### In the Modulation of Mood

Vagal signaling to the brain in response to immune stimuli contributes to the modulation of mood. Oral administration of the common bacterial enteropathogen *C. jejuni* in mice led to activation of the NTS, the central vagal afferent terminals, as well as brain regions regulating affective behaviors, such as the hypothalamic PVN, amygdala, and stria terminali (Gaykema et al., 2004). Furthermore, oral challenge with *C. jejuni* or *C. rodentium* induced anxiety-like behavior *via* activation of vagal pathways and brain areas, including PVN, amygdala and stria terminalis (Lyte et al., 2006; Goehler et al., 2008). This bacterial activation of vagal afferents may be *via* LPS and cytokines, as LPS and IL-1 have been shown to depress social exploration behavior, i.e., increase anxiety, *via* a vagal afferent pathway.

In functional dyspepsia, increased degranulation of duodenal mast cells are associated with anxiety and depression (Yuan et al., 2015). Furthermore, a 10-year follow-up study found that duodenal eosinophilia is associated with anxiety at follow-up (Ronkainen et al., 2021), suggesting gastroduodenal inflammation may contribute to psychological disorders in those patients who have gut symptoms preceding psychological disorders. Therefore, it is possible that inflammation and the increased vagal signaling in response to the inflammation contributes to the increase in psychological disorders. However, direct evidence is required to confirm this.

#### In the Modulation of Gastroduodenal Inflammation

The vagal afferent sensing of gastroduodenal immune signals leads to an anti-inflammatory effect. Indeed, stimulation of the vagus nerve, and specifically vagal afferent nerve, attenuated the systemic inflammatory response to endotoxin (Borovikova et al., 2000; Murray et al., 2021b) and, conversely, vagotomy sensitized animals to inflammation in a colitis animal model (Ghia et al., 2006). This anti-inflammatory effect is mediated by two pathways, the HPA axis and the vagal efferent pathway (Bonaz et al., 2016). Vagal afferents send inflammatory signals to the NTS. The NTS has projections to CRF containing neurons in the hypothalamus (Bonaz et al., 2016). This activates the HPA axis, the classic hormonal anti-inflammatory pathway, and the release of glucocorticoids to inhibit peripheral inflammation (Gaykema et al., 1995). The vagal efferent pathway is *via* a vagovagal inflammatory reflex, where vagal afferent mediated inflammatory signals can be centrally integrated and sent back *via* a descending vagal efferent pathway. The vagal efferent released neurotransmitter acetylcholine has been shown to attenuate the release of proinflammatory cytokines therefore inhibiting peripheral inflammation (Borovikova et al., 2000). This is in line with the increased hypothalamic CRF levels and plasma corticosterone levels in gastric irritation-induced functional dyspepsia rats (Liu et al., 2011), suggesting the gut inflammation-induced anti-inflammatory HPA axis pathway may be increased in functional dyspepsia. Whether the vagovagal inflammatory reflex is altered due to increased vagal afferent immune signals is not known. However, considering the generally reduced vagal efferent activity in functional dyspepsia, it is possible the vagovagal inflammatory reflex is impaired. This requires further investigation.

#### In the Modulation of Pain and Food Intake

Immune factors LPS, IL-1β, and TNF-α have been found to induce pain sensation and inhibit food intake *via* a vagal afferent pathway (Watkins et al., 1994, 1995; Schwartz et al., 1997). In *H. pylori* negative functional dyspepsia patients, peripheral blood mononuclear cell mediated release of cytokines IL-1β, IL-10, IL-6, and TNF-α were correlated with intensity of pain and cramps, and also nausea, vomiting and delayed gastric emptying, which can lead to reduced food intake (Liebregts et al., 2011). This suggests enhanced vagal signaling in response to inflammation may contribute to increased pain sensation and inhibition of food intake in functional dyspepsia.

## Vagal Efferent Signaling From the Brain to the Gut in Functional Dyspepsia

Vagal efferents transfer brain signals to the gut, including brain signals triggered by vagal afferent gut signals, circulating hormonal signals, or descending brain signals from other brain regions. These efferents, play an important role in the modulation of gastroduodenal motility, secretion and inflammation.

### Reduced Vagal Efferent Signaling in Functional Dyspepsia

Although there is no direct assessment of gastroduodenal vagal efferent activity in functional dyspepsia, a reduced cardiac vagal tone has been consistently observed in functional dyspepsia patients at basal level, after mental stress or after food intake (Hausken et al., 1993; Hveem et al., 1998; Lorena et al., 2002; Dal et al., 2014; Guo et al., 2018). The cardiac vagal tone was assessed based on variability in heart rate, including the respiratory sinus arrhythmia, monitoring the peak-to trough changes of heart rate within successive respiratory cycles, or 24-hour heart rate variability, monitoring the variation in the time interval between successive heartbeats over 24-hour period. Gastroduodenal vagal efferents share the same central origin with the cardiac vagal efferents (Bieger and Hopkins, 1987), and therefore the reduced cardiac vagal tone may more generally apply to the gastrointestinal tract. This is supported by evidence that reduced cardiac vagal tone is associated with impaired gastric relaxation and contraction in functional dyspepsia. In addition, increasing vagal tone with the use of breathing exercises reverses the impaired gastroduodenal functions in functional dyspepsia (Hjelland et al., 2007).

The reduced vagal efferent activity in functional dyspepsia could be due to the altered brain signals generated by stress. In functional dyspepsia patients, vagal tone is reduced after mental stress (Hausken et al., 1993; Hveem et al., 1998), and psychological factors explain a substantial amount of the reduction in vagal tone (Haug et al., 1994). CRF released from the hypothalamus is an important stress mediator and CRF in the brain inhibits vagal efferent activity (Broccardo and Improta, 1990; Kosoyan et al., 1999). Functional dyspepsia is associated with increased hypothalamic CRF levels (Zhu et al., 2020), which may contribute to the reduced vagal efferent activity. Furthermore, hypothalamic CRF levels can also be increased by gastric inflammation (Liu et al., 2011), suggesting reduced vagal efferent activity in functional dyspepsia could be mediated by gut inflammatory signals as well.

Functional dyspepsia is associated with an impaired vagovagal reflex in response to gut mechanical and chemical signals, as described above. Therefore, the reduced vagal efferent activity in functional dyspepsia could also be due to the impaired vagovagal reflex. The underlying mechanisms of the impaired vagovagal reflex are not known. However, in functional dyspepsia patients, reduced vagal efferent activity is correlated with increased sensation to small intestinal distension (Holtmann et al., 1998), therefore, the reduced vagal efferent activity is possibly due to a desensitization in response to the heightened vagal afferent signals. Furthermore, it is not known whether the vagal efferent anatomical innervation of the gut is altered in functional dyspepsia, which may also affect the vagal efferent function.

### Pathophysiological Roles of Reduced Vagal Efferent Signaling in Functional Dyspepsia

#### In the Modulation of Gastrointestinal Motility

Vagal efferents coordinate with the motor neurons of the enteric nerve system, continuously adjusting gastric smooth muscle relaxation and contraction to finely control gastric motility, which is essential for normal gastric accommodation, grinding, digestion and emptying. Two parallel vagal efferent pathways are involved in this function, namely the inhibitory and excitatory vagal motor pathways. Activation of the inhibitory vagal motor pathway, or inhibition of the excitatory vagal motor pathway (tonic contraction) results in a decrease in gastric contractile tone and intragastric pressure, gastric relaxation and an increase in the gastric reservoir. In contrast, activation of the excitatory vagal motor pathway, results in an increase in gastric contractile tone and gastric motility, a reduction in the gastric reservoir, and an increase in intragastric pressure. Therefore, inhibition of the inhibitory vagal motor pathway may contribute to the resting tonic contraction.

In functional dyspepsia patients, reduced vagal efferent activity is associated with delayed gastric emptying and antral hypomotility (Hausken et al., 1993; Haug et al., 1994; Guo et al., 2018). In addition, vagal activation by sham feeding and breath exercises improves antral motility, gastric emptying and gastric accommodation in functional dyspepsia patients (Hjelland et al., 2007; Lunding et al., 2008; Frokjaer et al., 2016). Therefore, vagal efferent activity could be an important mechanism and therapeutic target for altered gastroduodenal motility in functional dyspepsia. The main neurotransmitters used by the NTS neurons are acetylcholine, γ-aminobutyric acid (GABA), glutamate and noradrenaline, among which, GABA plays a major role in the DMV, where vagal efferent neurons are located (Travagli and Anselmi, 2016). However, evidence indicates GABA concentrations in multiple brain regions including DMV are not altered (Mak et al., 2020). However, it is unknown whether the functional effect of GABA on vagal efferent signaling is altered. In functional dyspepsia patients, abnormal brain signaling in response to gastric distension has been observed, including impaired pACC activation, and impaired deactivation of dorsal pons and amygdala (Van Oudenhove et al., 2010). The dorsal vagal complex receives projections from many brain regions, including cortex, hypothalamus, and amygdala (van der Kooy et al., 1984). Therefore, it is possible the brain signals received in the dorsal vagal complex are altered, leading to the impaired vagal efferent activity in functional dyspepsia, however, this requires further investigation. In functional dyspepsia patients, reduced vagal efferent activity is correlated with increased sensation to small intestinal distension, and partial vagotomy further increases the small intestinal sensation compared to patients without vagotomy, suggesting a role of impaired vagal efferent function in the development of gastrointestinal hypersensitivity (Holtmann et al., 1998). This could be an indirect effect due to the impact of impaired vagal efferent function on gastroduodenal motility, however, this requires further investigation.

#### In the Modulation of Gastrointestinal Secretion

Vagal efferents play an important role in the regulation of gastroduodenal secretions. Activation of vagal efferents result in the gastric secretion of acid from parietal cells, histamine from EC-like cells and gastrin from antral gastrin cells, as well as inhibition of somatostatin release from delta cells (Chang et al., 2003). In addition, vagal efferents stimulate bicarbonate secretion from the gastric and duodenal mucosa, which is important for neutralization of acid in the stomach and duodenum, protecting the mucosa from gastric acid injury (Fandriks and Jonson, 1990).

In functional dyspepsia, gastric acid levels are not different compared to healthy control (Collen and Loebenberg, 1989), and the mean plasma gastrin levels (at baseline, after exposing to acute stress and after food stimulation) are comparable to healthy control (Jonsson et al., 1998). However, histamine levels are higher in duodenal tissue of functional dyspepsia patients compared to healthy control (Zhu et al., 2020). Stress upregulated plasma somatostatin levels is earlier in functional dyspepsia patients compared to healthy controls, and the mean plasma somatostatin levels (at baseline, after exposing to acute stress and after food stimulation) are higher in patients with a higher degree of dyspeptic symptoms compared with patients with a lower degree of symptoms (Jonsson et al., 1998). Whether vagal efferents play a role in altering gastrointestinal secretions is not known and requires further investigation.

#### In the Modulation of Gastrointestinal Inflammation

Vagal efferents play a crucial anti-inflammatory role by transferring descending brain signals to modulate peripheral inflammatory responses. Activation of vagal efferents leads to release of acetylcholine from vagal efferent endings. Evidence indicates different types of immune cells may be sensitive to acetylcholine and modulated by vagal efferents, including macrophages, T-cells, and mast cells. *In vitro*, acetylcholine significantly inhibits the release of proinflammatory cytokines (e.g., TNF, IL-1β, IL-6, and IL-18) from endotoxin LPS stimulated macrophages, attenuating the peripheral inflammatory response to endotoxin (Borovikova et al., 2000). The acetylcholine inhibited TNF release from macrophages is mediated *via* activation of α-7 nicotinic Ach receptors (α7nAChRs) on macrophages (Wang et al., 2003). Anterograde tracing studies indicate vagal efferent fibers projecting to the small intestine are confined within the myenteric plexus regions, specifically around nNOS, VIP, and ChAT positive enteric neurons, which are in close proximity to α7nAChR of macrophages (Cailotto et al., 2014). Therefore, vagal efferents may modulate macrophages indirectly *via* the myenteric plexus. Recent findings indicate this activation of macrophages relies on acetylcholine released from T-cells activated by vagal efferent signaling (Murray et al., 2021a). Mast cells also express α7nAChRs (Sudheer et al., 2006). Acetylcholine stimulates rat mast cell degranulation and release of histamine (Masini et al., 1985a). The response of mast cells to acetylcholine is correlated with the sensitization of host and mast cells (Masini et al., 1985a,b). Furthermore, cervical vagal nerve stimulation was shown to increase histamine content in intestinal mucosal mast cells (Gottwald et al., 1995), and truncal vagotomy reduced the number of intestinal mucosal mast cells (Gottwald et al., 1997). This evidence suggests intestinal mast cells can be modulated by vagal efferent signaling from the brain, however, the role of mast cells in the anti-inflammatory role of vagal efferents needs further investigation.

In functional dyspepsia, reduced vagal efferent activity may lead to an impaired vagal efferent anti-inflammatory response, which may partly contribute to the increased inflammation in functional dyspepsia. Furthermore, in functional dyspepsia patients, vagal nerve stimulation increased vagal efferent activity and reduced plasma cytokine levels (Hou et al., 2020), suggesting that upregulating vagal efferent activity in functional dyspepsia could be a therapeutic target to improve inflammation.

## Therapeutic Implications of Vagus Nerve for Functional Dyspepsia

**FIGURE 3:**
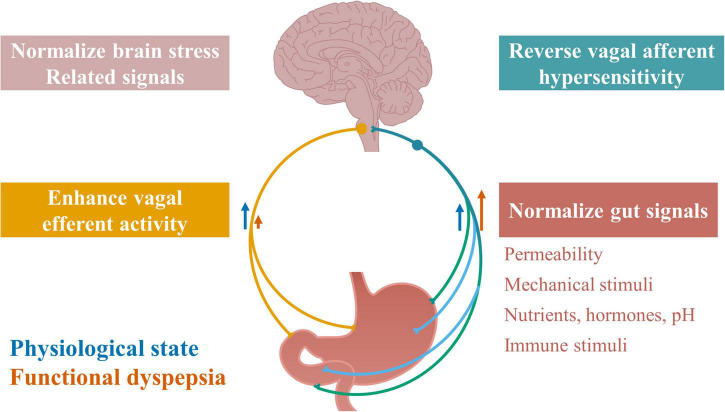
Therapeutic targets along vagal signaling pathways in functional dyspepsia. Restoring normal vagal signaling could be an important therapeutic target for functional dyspepsia, by normalizing any component in the vagal signaling loop, including normalizing gut signals, reversing vagal afferent hypersensitivity, normalizing brain stress related signals, and enhancing vagal efferent activity. Normalizing gut signals may target gastroduodenal permeability, mechanical stimuli, and chemical stimuli including nutrients, hormones, pH levels, and immune stimuli including microbiota.

Considering the potential pathophysiological roles of increased vagal afferent signaling and reduced vagal efferent signaling in functional dyspepsia, restoring normal vagal signaling could be an important therapeutic target for the subgroup of functional dyspepsia patients with altered vagal signaling. The approaches could target any components in the bidirectional signaling loop, including altered gut signals, vagal afferent hypersensitivity, altered brain signals and the reduced vagal efferent activity, as illustrated in Figure 3. Among them, increasing vagal efferent activity has been studied for the treatment of functional dyspepsia (Hjelland et al., 2007; Lunding et al., 2008; Zhu et al., 2021). Many approaches have been found to improve vagal efferent activity, including vagal nerve stimulation, gum chewing, meditation, deep breathing, physical exercise, and drugs targeting the cholinergic system. Vagal nerve stimulation has been shown to prevent an increase in intestinal permeability (Costantini et al., 2010; Zhou et al., 2013), promote gastric emptying by increasing pyloric sphincter relaxation, increase antral contraction amplitude and peristaltic velocity (Lu et al., 2018), and inhibit inflammation (Murray et al., 2021b). In functional dyspepsia patients, vagal nerve stimulation increased gastric accommodation and motility, reduced feeling of fullness, reduced bloating and pain, and reduced anxiety and depression scores *via* enhanced vagal efferent activity (Zhu et al., 2021). Similarly, sham feeding and deep breath exercises, known to increase vagal efferent activity, improved gastric accommodation and increased gastric motility, as well as increased the quality of life of functional dyspepsia patients (Hjelland et al., 2007; Lunding et al., 2008).

Alterations in vagal signaling might be part of the mechanisms of existing clinical approaches for the treatment of functional dyspepsia. Some prokinetic agents, such as acotiamide, have shown beneficial effects on improving gastric emptying and gastric accommodation in functional dyspepsia patients (Nakamura et al., 2017). These changes in gastrointestinal motility are likely to impact on the mechanical stimuli applied to the vagal afferents and thus their activity. Acid suppressive therapy is effective to improve heart burn and epigastric pain in a subset of functional dyspepsia patients with EPS (Moayyedi et al., 2004), likely *via* a reduction in acid-induced activation of vagal pain signals. *H. pylori* eradication in functional dyspepsia led to long-term relief of symptoms in functional dyspepsia patients with *H. pylori* infection (Malfertheiner et al., 2003), and is likely through elimination of infection associated mediators. Psychological therapy has also been shown to reduce symptoms, for up to a year, in a subset of functional dyspepsia patients and likely works by reducing stress related signals from modulating the vagal efferent pathway (Van Oudenhove and Aziz, 2013). Functional dyspepsia is a heterogeneous disorder with diverse symptoms and pathophysiological changes. No existing therapy is effective to the majority of patients. It is suggested that future therapies, for functional dyspepsia patients need to be personalized and based on the individual pathological subtypes (Talley, 2020). Altered vagal signaling may be a pathophysiological change in a subset of functional dyspepsia patients and, therefore, the vagus nerve could be a potential therapeutic target for this subset of patients. Targeting ascending vagal signals might be appropriate for pathologies associated with early satiety and pain, while targeting descending vagal signals might be more appropriate for pathologies such as inflammation and abnormal gastroduodenal motility. Further understanding of the underlying mechanisms responsible for altered vagal signaling in functional dyspepsia is needed to provide insight for the development of new therapeutics for the treatment of functional dyspepsia.

## Discussion

The vagus nerve plays a crucial role in the physiological modulation of brain and gut functions. In functional dyspepsia, vagal signaling between gut and brain may be disrupted in a subset of patients, contributing to a disrupted gut–brain axis and subsequent pathophysiological changes. However, the existing knowledge of this role is still very limited. A thorough understanding on the role of the vagus nerve in functional dyspepsia may provide potential clinical pathophysiological targets and eventually therapeutic targets for this disorder. This requires further studies into the mechanisms driving disordered vagal signaling including the: (1) molecular mechanisms driving the altered vagal signaling in functional dyspepsia (impaired vagal efferent signaling and heightened mechanosensitivity of vagal afferents); (2) role of chemosensitive vagal afferents in functional dyspepsia (response to local gastroduodenal chemical changes, including pH, immune associated factors, nutrients and hormones); (3) anatomical changes of vagal nerve in functional dyspepsia; (4) association of vagal signaling pathways to functional dyspepsia symptoms and pathophysiologies; and (5) effectiveness of reversing altered vagal signaling in improving functional dyspepsia symptoms and associated pathophysiological changes, etc. These potential studies will rely on clinical research and, importantly, the animal models of functional dyspepsia. Over the last decade, many animal models of functional dyspepsia have been developed, including stress-related models and gastrointestinal irritation-induced animal models, which aim to mimic the bidirectional origin of this disorder. However, none of these animal models are widely used and accepted, and the validity of these models is still not clear, as discussed in recent reviews (Ye et al., 2018; Accarie and Vanuytsel, 2020). Therefore, further understanding of the animal models is essential when choosing the appropriate animal model for studying the role of the vagus nerve, as well as other mechanisms of functional dyspepsia.

## Author Contributions

HL wrote the review under the supervision of AP. AP edited the manuscript and provided assistance with the structure of the review. Both authors contributed to the manuscript and approved the submitted version.

## Conflict of Interest

The authors declare that the research was conducted in the absence of any commercial or financial relationships that could be construed as a potential conflict of interest.

## Publisher’s Note

All claims expressed in this article are solely those of the authors and do not necessarily represent those of their affiliated organizations, or those of the publisher, the editors and the reviewers. Any product that may be evaluated in this article, or claim that may be made by its manufacturer, is not guaranteed or endorsed by the publisher.

## Abbreviations

IGLEs: intraganglionic laminar endings
IMAs: intramuscular arrays
NTS: nucleus of solitary tract
DMV: dorsal motor nucleus of the vagus
CCK: cholecystokinin
LPS: lipopolysaccharide
AgRP: agouti-related protein
PVN: paraventricular nucleus
PDS: postprandial distress syndrome
CRF: corticotrophin-releasing factor
BDNF: brain-derived neurotrophic factor
EPS: epigastric pain syndrome
PAG: periaqueductal gray
GLP-1: glucagon-like peptide-1
PYY: peptide YY
EC: enterochromaffin
TNF: tumor necrosis factor
IL-1β: interleukin-1β
HPA axis: hypothalamic–pituitary–adrenal axis
*H. pylori*: *Helicobacter pylori*
*C. jejuni*: *Campylobacter jejuni*
*C. rodentium*: *Citrobacter rodentium*
α7nAChRs: α-7 nicotinic Ach receptors.
